# Suppression of premature ventricular complexes with the PDE5 inhibitor sildenafil: First clinical experience

**DOI:** 10.1113/EP092932

**Published:** 2025-09-10

**Authors:** David C. Hutchings, Christopher P. Denton, Luigi Venetucci, Andrew W. Trafford

**Affiliations:** ^1^ Unit of Cardiac Physiology, Division of Cardiovascular Sciences, Faculty of Biology, Medicine and Health, Manchester Academic Health Sciences Centre, 3.24 Core Technology Facility University of Manchester Manchester UK; ^2^ Centre for Rheumatology, Royal Free Campus, NW3 2PS University College London London UK

**Keywords:** arrhythmia, phosphodiesterase‐5, sildenafil

## Abstract

The phosphodiesterase‐5 inhibitor sildenafil suppresses ventricular arrhythmias in a sheep model of drug‐induced long QT. In that study, ventricular arrhythmias were abolished by reducing premature ventricular complexes (PVCs) and delaying PVC onset, thus preventing ‘R‐on‐T’ ventricular tachycardia. Evidence for effects in humans with arrhythmias is lacking. In this case study, a 50‐year‐old female with a history of PVCs and systemic sclerosis was started on sildenafil for Raynaud's phenomenon in line with current treatment recommendations. During initiation, the patient wore a 7‐day cardiac monitor. Two subtypes of PVCs were observed: one distinct morphology arising <400 ms from the preceding sinus beat (‘early’) and a separate morphology >400 ms from the preceding sinus beat (‘late’). Sildenafil abolished late PVCs and substantially reduced the frequency of early PVCs. Of those early PVCs remaining during sildenafil treatment, PVCs arose later in the cardiac cycle, 21 ms further from the preceding T wave apex. During washout, PVCs returned in frequency and timing towards baseline values. We report the first case suggesting an anti‐arrhythmic property of sildenafil in a human.

## Abstract (highlights)

- **What is the main observation in this case?**
  Initiation of the phosphodiesterase‐5 inhibitor sildenafil in a patient with Raynaud's phenomenon substantially reduced the frequency of premature ventricular complexes (PVCs). Of the PVCs remaining during sildenafil treatment, their timing was delayed, arising further from the preceding T wave. The effects of sildenafil were reversed with washout.
- **What insights does it reveal?**
  The effect of sildenafil on PVCs in this case provides first‐in‐human evidence suggesting an anti‐arrhythmic property of sildenafil.

## 1 INTRODUCTION

Ventricular arrhythmias frequently arise in the setting of β‐adrenergic stimulation (Priori et al., 1988). Catecholamines stimulate the β‐adrenoceptor in cardiac myocytes to activate protein kinase A, leading to sarcoplasmic reticulum (SR) Ca^2+^ loading via Ca^2+^ entry through L‐type Ca^2+^ channels and SR Ca^2+^ uptake by SERCA. When SR Ca^2+^ reaches a threshold level, it releases Ca^2+^ spontaneously, giving rise to pro‐arrhythmic Ca^2+^ waves and thence after‐depolarizations and triggered arrhythmias (Ferrier et al., 1973). Antagonizing β‐adrenergic stimulation with β‐adrenoceptor blockers is a mainstay anti‐arrhythmic strategy (Priori et al., 2015), but their initiation and up‐titration are often limited by negative inotropy, bradycardia and other side effects, including fatigue, poor peripheral perfusion, erectile dysfunction and bronchospasm. Novel alternatives would be valuable.

An alternative anti‐arrhythmic strategy is to counteract downstream β‐adrenergic signalling by activating protein kinase G in cardiac myocytes (Takimoto et al., 2005). The PDE5 inhibitor sildenafil activates protein kinase G by preventing the breakdown of cGMP in cardiac myocytes. We previously reported sildenafil suppression of ventricular arrhythmias in a sheep model of drug‐induced long QT (Hutchings et al., 2021). In that study, ventricular arrhythmias were abolished by a reduced incidence of premature ventricular complexes (PVCs) and delayed timing of PVCs, thus preventing ‘R‐on‐T’ events. At the cellular level, sildenafil reduced Ca^2+^ entry and reduced Ca^2+^ uptake into the SR, thus reducing the frequency of after‐depolarizations and delaying their onset (Hutchings et al., 2022).

Sildenafil is widely used in the treatment of erectile dysfunction, pulmonary hypertension and Raynaud's phenomenon. In addition to its proven safety profile, accumulating evidence suggests that it is cardioprotective, being associated with reduced cardiovascular mortality (Anderson et al., 2016, 2017) and improving systolic function in animal models of heart failure with reduced ejection fraction (Lawless et al., 2019; Nagayama et al., 2009). Here, we report a case of systemic sclerosis treated with sildenafil for Raynaud's phenomenon, with apparent improvement in PVCs, suggesting a potential anti‐arrhythmic property in humans.

## 2 PARTICIPANT INFORMATION

Written consent was provided by the patient for publication of this report. The data that support the findings of this study are available upon reasonable request. A 50‐year‐old white female with systemic sclerosis described severe painful secondary Raynaud's phenomenon and was started on sildenafil in line with current treatment recommendations for this complication (Denton et al., 2024). She had initially tried nifedipine but was unable to tolerate this owing to symptomatic low blood pressure. Her past medical history included lymphoma successfully treated with chemotherapy (rituximab and bendamustine), achieving full remission. She previously had ectopic beats detected on a previous Holter ECG. Recent echocardiography revealed normal biventricular size and systolic function, with no valve disease.

Before initiation of sildenafil, the patient's heart rhythm was monitored using a wearable 7‐day cardiac monitor (Life Signals 1AXE, CE marked and FDA approved), placed over the left anterior chest wall. After 2 days of control recordings, she commenced sildenafil at 25 mg once daily. After 3 days of sildenafil, she opted to stop the sildenafil to assess whether its effects were reversible, and her heart was monitored for a further 2 days (sildenafil half‐life ∼4 h). Subsequently, over a period of 3 weeks, her sildenafil dose was gradually increased to a maximum of 50 mg three times daily on the advice of her rheumatologist. At this point, her heart was monitored for a further 4 days with a second monitor. Recordings were analysed by a consultant cardiologist (D.C.H.). Throughout the recordings, the patient performed her routine level of activity and consumed the same amount of tea and coffee on each day. She drank minimal alcohol during the period. Shortly after finishing the second recording period, the patient stopped taking sildenafil, owing to headaches and insufficient control of her Raynaud's phenomenon.

Data are presented as the mean ± SD. ECG traces were analysed using LabChart Pro, v.7 (AD instruments). Data analysis was performed using GraphPad Prism, v.9. Normality of data distribution was tested using a D'Agostino‐Pearson or Shapiro–Wilk test. For normally distributed data, differences between treatment groups were determined using Student's unpaired *t*‐test, and non‐parametric tests were used when data were not normally distributed, as indicated in the figure legends. Differences were considered statistically significant at *p* < 0.05.

## 3 FINDINGS

**FIGURE 1:**
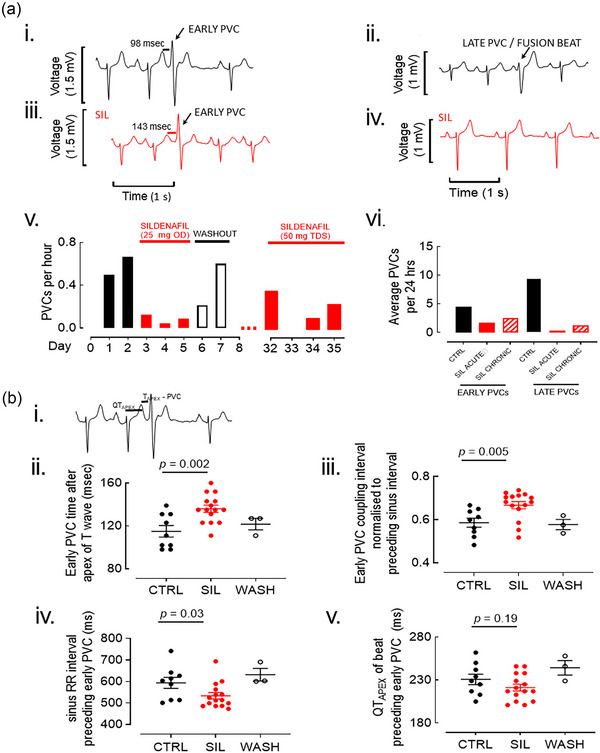
Effect of sildenafil on premature ventricular complexes (PVCs). a) Representative surface ECG recordings of an early PVC (i) and late PVC (ii). The effect of sildenafil is shown for early PVC (iii, delayed relative to preceding T wave) and late PVC (iv, abolished). (v) Daily frequency of PVCs and vi) PVC summary mean data. Total PVCs in CTRL *n* = 28, SIL 25 mg OD *n* = 6, washout *n* = 8, SIL 50 TDS *n* = 15. Washout on day 7 was for 5 h (recording terminated after this). (b) Analysis of the timing of early PVCs in control, sildenafil (includes both 25 mg OD and 50 mg TDS groups) and washout. (i) Example analysis of interval between Q wave and TAPEX (QTAPEX) in the sinus beat immediately preceding an early PVC, and interval between TAPEX and early PVC (TAPEX‐PVC). (ii) Mean data for early PVC timing after apex of T wave. Student's unpaired *t*‐test. (iii) Mean data for PVC coupling interval normalized to the preceding sinus RR interval. Mann–Whitney *U*‐test. (iv) Mean data for the sinus RR intervals preceding early PVCs. Mann–Whitney *U*‐test. (v) Mean data for the QTAPEX in sinus beats immediately preceding an early PVC. Student's unpaired *t*‐test. For each analysis, CTRL early PVCs *n* = 9, SIL early PVCs *n* = 15, washout PVCs *n* = 3. Abbreviations: CTRL, control; PVC, premature ventricular complex; SIL, sildenafil; OD, once daily; TDS, three times daily.

Of the total 264 h of recording, 261 h were analysed, with the remainder discarded owing to noise. At baseline, the patient had 14.0 ± 2.8 PVCs/24 h (range 12–16; Figure 1ai,ii,v, days 1 and 2). The vast majority of PVCs arose during daytime hours (89% between 08.00 and 20.00 h) and at higher heart rates (92 beats min^−1^ vs. the average of 78 beats min^−1^). None of the PVCs arose on the preceding T wave (‘R‐on‐T’), and there were no couplets or non‐sustained ventricular tachycardia.

Two subtypes of PVCs were observed. PVCs with a single morphology were observed early in the cardiac cycle, <400 ms from the preceding sinus beat (345 ± 27 ms from preceding beat; Figure 1ai) and are classified as ‘early’. PVCs with a different morphology were seen arising much later in the cardiac cycle (632 ± 105 ms from preceding beat), with some merging with subsequent sinus beats to form ‘fusion beats’ (Figure 1aii). These are classified as ‘late’ (arising >400 ms from the preceding sinus beat). The two subtypes of PVCs were considered to arise from different sites in the ventricles and were therefore analysed separately.

**FIGURE 2:**
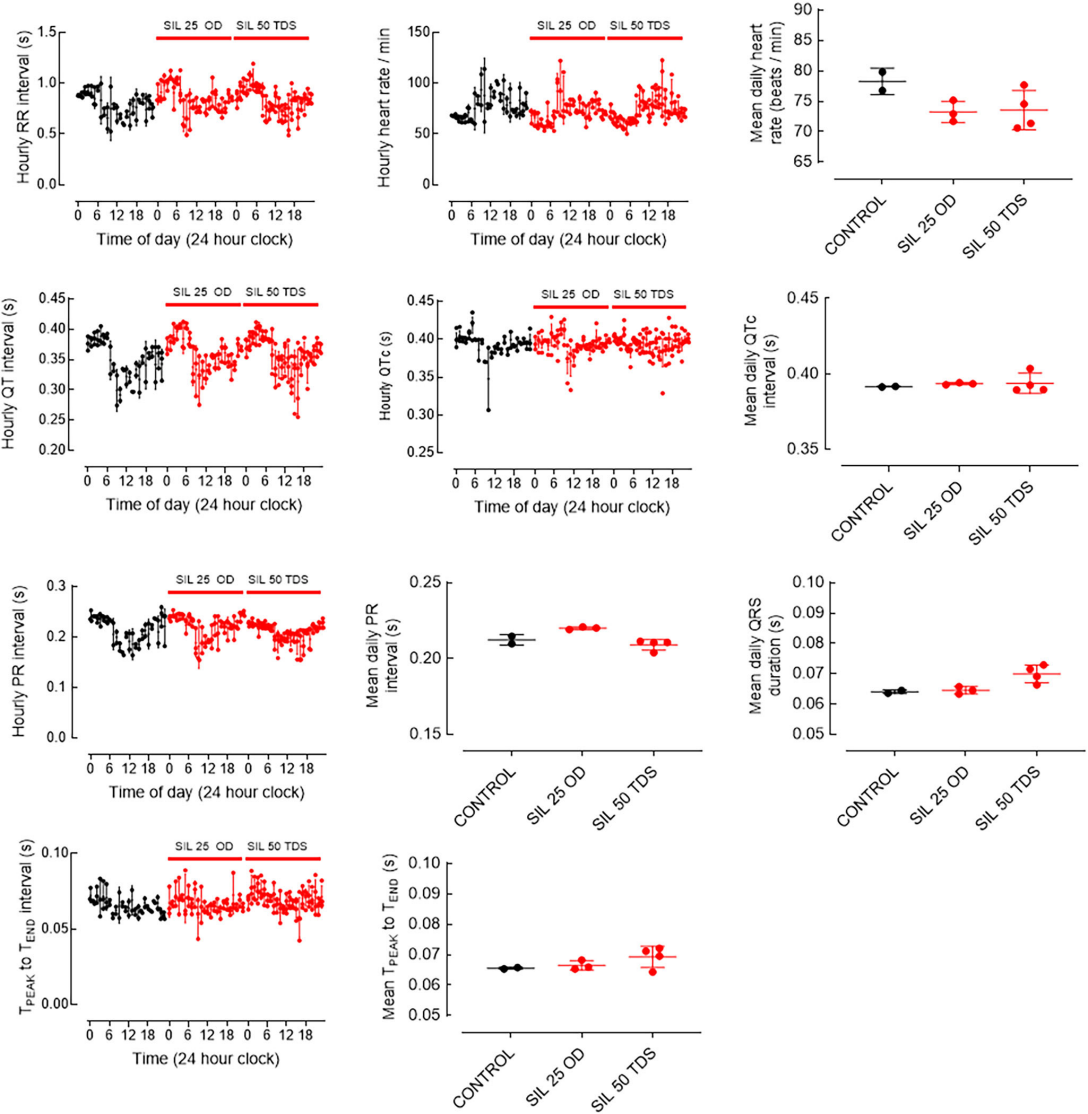
Sildenafil effect on hourly and mean ECG parameters. For hourly graphs, each data point represents a 10‐beat average at the start of each hour. Error bars show the mean ± SD. For daily mean graphs, each data point represents a daily average of hourly readings. Control recording is 2 days, Sil 25 mg OD is 3 days, and SIL 50 mg TDS is 4 days. Abbreviations: CTRL, control; SIL, sildenafil.

The daily effect of sildenafil on PVCs can be observed in Figure 1av, and the mean effects are summarized in Figure 1avi. Acute sildenafil (days 3–5) nearly abolished late PVCs (from 9.5 ± 4.9 to 0.3 ± 0.6 day^−1^; Figure 1a iv & vi) and reduced the frequency of early PVCs (from 4.5 ± 2.1 to 1.7 ± 1.2 day^−1^). During the washout period (days 6–8), PVCs of both types increased towards baseline levels. The effect of sildenafil on average PVC frequency persisted in chronic treatment (Figure 1av, days 32–35), although there was considerable variation between days. Heart rate was slightly reduced during sildenafil treatment (Figure 2). Sildenafil appeared to have no effect on basic ECG parameters of PR, QTc, QRS or T_PEAK_–T_END_ intervals (Figure 2).

The character of early PVCs was modified by sildenafil. During sildenafil treatment, early PVCs occurred at higher heart rates than in control conditions (summary data in Figure 1biv). Sildenafil also altered the timing of early PVCs in relationship to the preceding T wave (Figure 1ai,iii; and summary data Figure 1bii,iii); during sildenafil treatment, early PVCs were on average 21 ms further from the T wave apex (T_APEX_) (136 ± 13 vs. 115 ± 16 ms after T_APEX_, *p* = 0.002). This could be attributable either to PVCs arising later in the cardiac cycle or to rate‐related shortening of the QT interval in the preceding beat. To establish which was responsible, the QT interval and time from the Q wave to T_APEX_ (QT_APEX_) were calculated for each beat preceding a PVC (Figure 1bi, for example). This revealed a small shortening of the QT_APEX_ (9.5 ms) during sildenafil treatment, which was not statistically significant (*p* = 0.19; Figure 1bv) and did not account fully for the effect of sildenafil on the later timing from T_APEX_.

**FIGURE 3:**
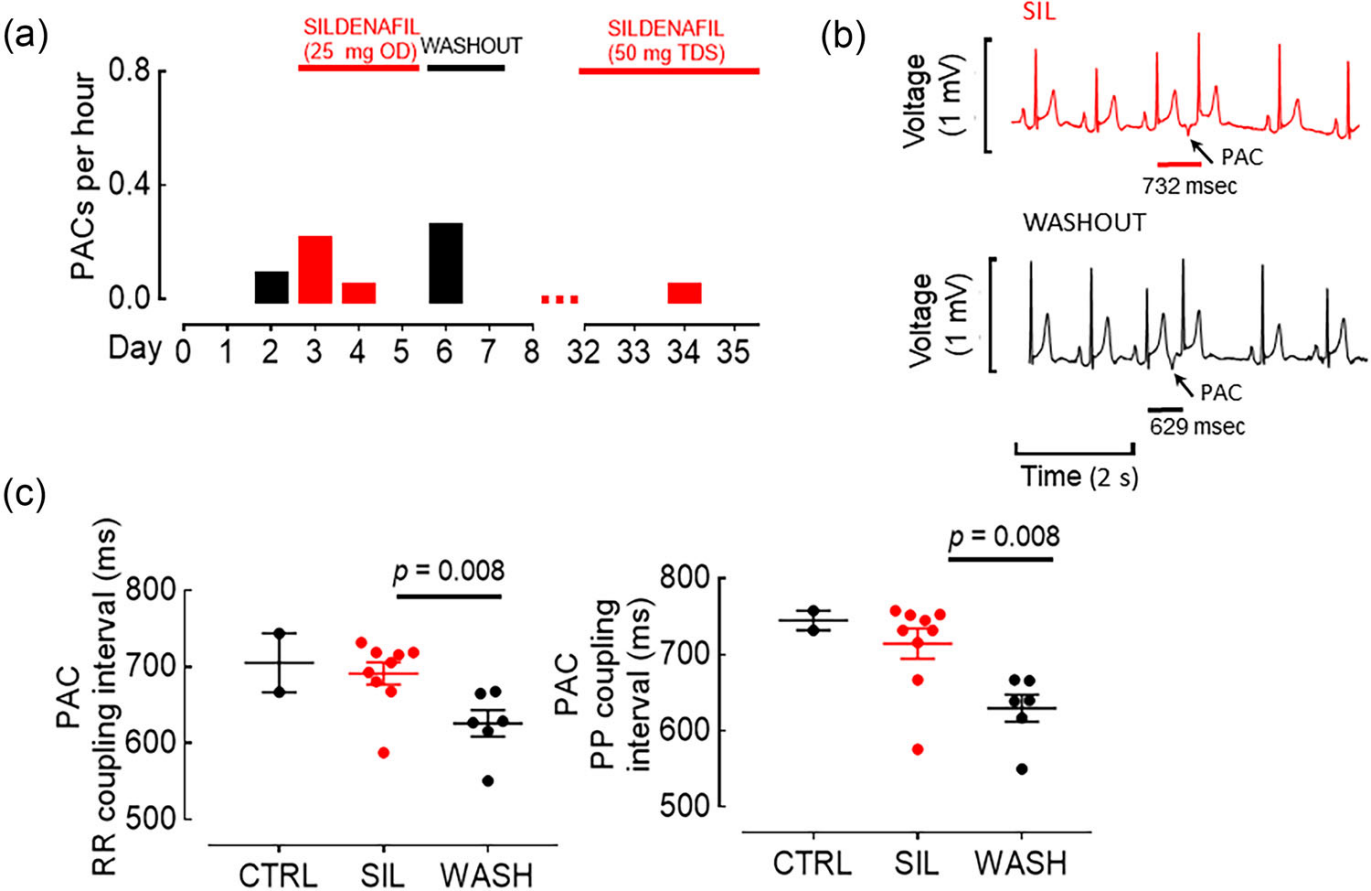
Effect of sildenafil on PACs. (a) Daily incidence of PACs. (b) Representative surface ECG recordings of PACs, in sildenafil (upper trace) and washout (lower trace) conditions. (c) Summary mean data on the PAC coupling intervals for RR interval between preceding sinus beat and PAC (left), and PP interval (right). For both, Mann–Whitney *U*‐test. For each analysis, CTRL early PACs *n* = 2, SIL early PACs *n* = 9, washout PACs *n* = 6. Abbreviations: CTRL, control; PAC, premature atrial complex; SIL, sildenafil.

Finally, premature atrial complexes (PACs) were also observed throughout the recording but were much less frequent (Figure 3a,b). The low baseline frequency of PACs makes it difficult to draw conclusions about the effect of sildenafil on their frequency. The PACs were most frequent during the washout period, and a comparison has been made between their coupling intervals (Figure 3b,c). Here, sildenafil PACs appear to arise later in the cardiac cycle compared with washout PACs.

## 4 DISCUSSION

We provide the first report of an anti‐arrhythmic effect of sildenafil in a human. Sildenafil reduced the frequency of early PVCs and abolished late PVCs. In addition, of the early PVCs remaining, sildenafil delayed their timing in the cardiac cycle, arising later after the T wave. These effects appeared to be reversed with a 2‐day washout (sildenafil half‐life ∼4 h).

This report is limited to observations in one individual and, therefore, concrete conclusions cannot be drawn. Nevertheless, the same properties of substantially reduced PVCs, combined with a delay in their onset, were observed robustly in a pro‐arrhythmic sheep in vivo model of drug‐induced long QT syndrome (Hutchings et al., 2021), in which sildenafil suppressed ventricular arrhythmias by reducing the incidence of PVCs and delaying PVCs, thus preventing ‘R‐on‐T’ events. In that study, sildenafil had no effect on baseline surface ECG parameters; instead, anti‐arrhythmic properties were present at the intracellular level. Sildenafil reduced SR Ca^2+^ content (attributable to reduced SR Ca^2+^ uptake via SERCA and reduced cellular Ca^2+^ influx), which abolished spontaneous intracellular SR Ca^2+^ release and therefore suppressed after‐depolarizations. Suppression of cellular after‐depolarizations reduces PVCs. In addition, in cells where after‐depolarizations remained, the slower SR Ca^2+^ refilling delays after‐depolarizations such that they arise later in each cardiac cycle. This provides a possible mechanism for the sildenafil delayed timing of PVCs observed both in sheep and in this human study.

It is possible that sildenafil has the same effect in humans. Again, like that study, in this subject, sildenafil had no effect on baseline ECG parameters of PR, QT or QRS durations, suggesting that surface ion channel effects are not a primary mechanism.

During sildenafil treatment, PVCs arose at faster heart rates (shorter RR intervals), causing a very small non‐statistically significant decrease in QT_APEX_ immediately before the PVC. This was likely to be rate‐related shortening of the QT interval. Although this might have contributed to the later timing of PVCs from the T_APEX_, it is unlikely to be the main driver. No statistically significant effect of sildenafil was observed on the incidence of PACs, although their baseline frequency was too low to draw conclusions.

We identified two subtypes of PVCs with separate morphologies and timings (‘early’ and ‘late’), probably arising from separate sites in the ventricle. The effect of sildenafil differed between ‘early’ and ‘late’ PVCs. Sildenafil reduced the frequency of early PVCs and delayed the onset of those remaining. Sildenafil virtually abolished late PVCs, making it impossible to assess the timing effects of sildenafil in this group; it is possible that sildenafil delayed late beats such that the next sinus beat occurred before they could arise; however, this would need testing experimentally.

It is not clear why sildenafil had differing efficacy on different types of PVCs. It is interesting to speculate that different PVC types might have different underlying mechanisms; for example, her early PVCs might not be Ca^2+^ dependent (although sildenafil delayed their onset, which would argue against this). Alternatively, sildenafil might suppress PVCs by delaying them beyond the next sinus beat, and delaying an early PVC to that extent is harder to achieve than with a late PVC.

Although the frequency of PVCs in this patient is within normal limits for the general population (Kostis et al., 1981), arrhythmias are also recognized complications of scleroderma (Bissell et al., 2020, 2017) and might be caused by myocardial scarring, inflammation or ischaemia (Vacca et al., 2014). Additional to the direct electrophysiological effects of sildenafil, it is conceivable that sildenafil might suppress arrhythmias by modifying inflammatory pathways (di Luigi et al., 2016; Hutchings et al., 2018), as well as lowering blood pressure and vascular function (Vardi et al., 2002), and these are avenues of further study.

There are several limitations to this case study. There was no placebo control arm and no treatment randomization or blinding was performed. The PVC frequency was low at baseline (PVC frequency was within the top 20% of patients with structurally normal hearts; Kostis et al., 1981), and we cannot rule out the possibility that PVCs were reduced by chance (Raeder et al., 1988), given that the daily fluctuation of PVCs is well documented. However, the suppression we saw is greater than expected by chance alone (Raeder et al., 1988) and was reversible on cessation of the drug. Improved control of Raynaud's phenomenon could have contributed to the lower incidence of PVCs (e.g., reduced pain, hence reduced sympathetic activation) (Huskisson, 1974; Lampert et al., 2000). However, it is unlikely that these explain the effect on PVCs, because sildenafil provided minimal improvement in her Raynaud's phenomenon symptoms, and higher doses were accompanied by debilitating headaches such that she stopped the drug shortly after recordings ended. Finally, in contrast to the anti‐arrhythmic properties described here, it should be noted that there have been isolated reports of arrhythmias after sildenafil ingestion (Rasmussen et al., 2007; van der Hooft et al., 2004), although overwhelming epidemiological evidence indicates a proven safety profile (Anderson et al., 2016, 2017). In summary, this case study provides the first evidence of anti‐arrhythmic effects of sildenafil in humans. Further clinical studies would be required to confirm these findings.

## AUTHOR CONTRIBUTIONS

David C. Hutchings conceived of the work, performed data analysis and wrote the manuscript. Andrew W. Trafford, Christopher P. Denton and Luigi Venetucci interpreted the data, edited the manuscript and revised it critically for important intellectual content. All authors approved the final version of the manuscript and agree to be accountable for all aspects of the work in ensuring that questions related to the accuracy or integrity of any part of the work are appropriately investigated and resolved. All persons designated as authors qualify for authorship, and all those who qualify for authorship are listed.

## CONFLICT OF INTEREST

The authors have no conflicts to disclose.
